# Preliminary Evidence for the Impact of Combat Experiences on Gray Matter Volume of the Posterior Insula

**DOI:** 10.3389/fpsyg.2017.02151

**Published:** 2017-12-12

**Authors:** Ashley N. Clausen, Sandra A. Billinger, Jason-Flor V. Sisante, Hideo Suzuki, Robin L. Aupperle

**Affiliations:** ^1^Laureate Institute for Brain Research, Tulsa, OK, United States; ^2^Departments of Psychology and Community Medicine, University of Tulsa, Tulsa, OK, United States; ^3^Department of Psychology, University of Missouri-Kansas City, Kansas City, MO, United States; ^4^Department of Physical Therapy and Rehabilitation Science, University of Kansas Medical Center, Kansas City, KS, United States; ^5^University of Nebraska-Lincoln, Lincoln, NE, United States

**Keywords:** combat, veterans, flow-mediated dilation, cardiovascular, posterior insula

## Abstract

**Background:** Combat-exposed veteran populations are at an increased risk for developing cardiovascular disease. The anterior cingulate cortex (ACC) and insula have been implicated in both autonomic arousal to emotional stressors and homeostatic processes, which may contribute to cardiovascular dysfunction in combat veteran populations. The aim of the present study was to explore the intersecting relationships of combat experiences, rostral ACC and posterior insula volume, and cardiovascular health in a sample of combat veterans.

**Method:** Twenty-four male combat veterans completed clinical assessment of combat experiences and posttraumatic stress symptoms. Subjects completed a magnetic resonance imaging scan and autosegmentation using FreeSurfer was used to estimate regional gray matter volume (controlling for total gray matter volume) of the rostral ACC and posterior insula. Flow-mediated dilation (FMD) was conducted to assess cardiovascular health. Theil-sen robust regressions and Welch's analysis of variance were used to examine relationships of combat experiences and PTSD symptomology with (1) FMD and (2) regional gray matter volume.

**Results:** Increased combat experiences, deployment duration, and multiple deployments were related to smaller posterior insula volume. Combat experiences were marginally associated with poorer cardiovascular health. However, cardiovascular health was not related to rostral ACC or posterior insula volume.

**Conclusion:** The present study provides initial evidence for the relationships of combat experiences, deployment duration, and multiple deployments with smaller posterior insula volume. Results may suggest that veterans with increased combat experiences may exhibit more dysfunction regulating the autonomic nervous system, a key function of the posterior insula. However, the relationship between combat and cardiovascular health was not mediated by regional brain volume. Future research is warranted to further clarify the cardiovascular or functional impact of smaller posterior insula volume in combat veterans.

## Introduction

Cardiovascular (CV) disease continues to be the leading cause of mortality in the United States, affecting one in three adults (Hoyert, 2011; Heron and Anderson, 2016). Individuals who have experienced significant trauma (e.g., combat), as well as those diagnosed with PTSD, are at an increased risk for developing CV disease (Elder et al., 1997; Wentworth et al., 2013) and CV-related death (Boscarino, 2008). Recently, a prospective study conducted as part of the Millenium Cohort found that veterans who experienced combat were 1.6 times more likely than those without combat to develop CV disease (Crum-Cianflone et al., 2014). Interestingly, and in contrast to previous findings, PTSD was not related to CV disease after controlling for anxiety and depression (Crum-Cianflone et al., 2014) suggesting that combat experiences may play a unique role in the development of CV disease.

It has been proposed that prolonged hyperarousal, secondary to trauma exposure, may influence CV health through heightened sympathetic response and increased immune cell inflammation. This sympathetic response may then contribute to autonomic dysregulation, and/or increased activation of the renin-angiotensin hormonal system, which is implicated in regulating blood pressure (Brudey et al., 2015). PTSD has also been associated with dysregulation and volumetric changes in neural networks, particularly the salience network (connected by aspects of the anterior cingulate and insular cortex) that are linked with autonomic and cardiovascular regulation (Aupperle et al., 2012; Schmidt et al., 2015; Stillman and Aupperle, 2015). The salience network is implicated in detection of novel or emotionally-relevant stimuli (Menon, 2015). In the context of individuals exposed to trauma, there is evidence of increased (heightened awareness of interoceptive responses) and decreased (emotional detachment) activity within the salience network (Lanius et al., 2015). However, it is likely that dysfunction within interacting physiologic and neural systems contribute to poorer CV health, and eventually a diagnosis of CV disease, for individuals with PTSD (Wentworth et al., 2013; Brudey et al., 2015).

There are many potential contributors to poor CV health in trauma-exposed populations, which have been identified primarily through cross-sectional studies including increased levels of total cholesterol and low-density lipoprotein cholesterol, as well as significantly lower levels of high-density lipoprotein cholesterol (Solter et al., 2002). In addition, those with a specific diagnosis of PTSD have higher resting heart rate and blood pressure, as well as increased heart rate and blood pressure reactivity to emotional provocation (Cohen et al., 2000; Buckley et al., 2004), and are three times more likely to have atrioventricular conduction defects (decreased conduction throughout the heart such as the atria and ventricles), even after controlling for additional anxiety symptoms and depression (Boscarino and Chang, 1999) compared to non-PTSD controls.

In addition, a sub-set of literature has begun to examine endothelial function, a more direct measure of CV health in relation to trauma. Flow-mediated dilation (FMD) is a non-invasive measure of endothelial function. FMD assesses conduit vessel function using Doppler ultrasound technology to create a structural and functional images of the brachial artery (Guthikonda et al., 2007). FMD is used to determine the percent change in artery dilation from pre- to post-occlusion (Guthikonda et al., 2007), which has been shown to predict CV disease in older populations (Yeboah et al., 2007). One study reported that, when compared to those with moderate levels of PTSD severity, police officers with high levels of PTSD severity showed significantly poorer FMD response, reflecting poorer CV function, regardless of demographic and lifestyle factors (Violanti et al., 2006). Furthermore, older veteran populations (mean age 68.5) with PTSD were more likely to exhibit poorer FMD response relative to those without PTSD (Grenon et al., 2016). However, the use of older individuals with known CV disease limits the ability to determine if FMD response is a result of PTSD, age, or CV disease (Grenon et al., 2016). Expanding on this, we recently conducted a study to assess FMD in physically healthy combat veterans with varying levels of PTSD. While PTSD severity did not relate to FMD as in previous research, veterans in the study exhibited surprisingly impaired FMD overall (Clausen et al., 2016). Thus, poorer FMD response may be a by-product of increased trauma exposure or stress rather than the severity of PTSD. However, the replication regarding the impact of combat experiences on CV health in veteran populations is warranted.

The link between trauma exposure and CV disease may be influenced by differences or changes in brain morphology, although this has not yet been well-investigated. For example, detrimental effects of trauma exposure on brain volume within regions involved in autonomic processing could, in turn, contribute to poorer CV health (and/or vice versa). In fact, prior research suggests that both trauma exposure and PTSD may have unique and detrimental effects on gray matter (GM) volume, especially hippocampal volume, compared to non-trauma exposed controls (Woon et al., 2010) as well as reduced GM volume within the amygdala, which are implicated in affective and physiological responses specific to fear or threat-related stimuli (Davis and Whalen, 2001; Sergerie et al., 2008; Adolphs, 2010). While prior research supports dysfunction and volumetric changes within the salience network (Aupperle et al., 2012; Schmidt et al., 2015; Stillman and Aupperle, 2015), which includes the dorsal aspect of the anterior cingulate cortex (ACC) and anterior insula, there is also support for volumetric changes within the rostral ACC and posterior insula (for review, see Stillman and Aupperle, 2015). The rostral ACC is thought to regulate autonomic arousal in response to stress (Stevens et al., 2011). The posterior insula has extensive connectivity with the limbic system including the ACC (Oppenheimer et al., 1992), and is involved in processing visceral and homeostatic information, as well as physiological arousal (Craig, 2002). Additionally, activation of both the rostral ACC and posterior insula are associated with changes in autonomic responses during stressor tasks including alterations in heart rate and blood pressure (Oppenheimer et al., 1992; Critchley et al., 2003). Research indicates that neural correlates of CV regulation overlap with those regions impacted by PTSD, specifically within the rostral anterior cingulate and posterior insula (Critchley et al., 2000). Given that the rostral ACC and posterior insula are specifically implicated in regulating and monitoring physiological arousal, respectively, as opposed to emotional awareness and regulatulation (dorsal ACC and anterior insula), these regions may link combat experiences with CV disease.

The current study is the first to investigate the relationships of combat experiences with FMD, and brain volume in combat veterans. We aimed to determine the relationships of rostral ACC and posterior insula volume with (1) combat experiences and (2) FMD in a veteran population. We hypothesized that increased combat experiences, as well as poorer FMD response, would be related to smaller rostral ACC and posterior insula volume. Last, if rostral ACC or posterior insula volumes were associated with both combat experiences and FMD, we hypothesized that regional GM volume would mediate the relationship between combat experiences and FMD.

## Materials and methods

### Subjects

Subjects were recruited as a part of the study at the University of Missouri-Kansas City (PI: RLA), and included 24 male combat veterans ages of 18–55 (median age = 31.0, MAD = 5.9) with varying degrees of PTSD symptoms (Table 1) who served in combat since the onset of Operation Iraqi Freedom. Subjects completed a phone screen (self-report) and initial assessment to assess inclusion and exclusion criteria and were excluded from the current study if they were not a current or former member of the United States military, or if they reported any of the following: psychiatric symptoms requiring immediate attention, substance or alcohol abuse or dependence over the past 6 months, diagnosis of any medical condition that directly affects CV function (i.e., atherosclerosis), history of moderate to severe head injury (loss of consciousness > 30 min and/or post-traumatic amnesia > 1 day), history of neurological disorder, reported being diagnosed or met criteria for schizophrenia or bipolar I disorder (as determined via the Mini International Neuropsychiatric Inventory; Sheehan et al., 1998), or use of medications within the last 30 days that affect the CV response (e.g., antihypertensives). No subjects were being treated with medications used for sleep or antidepressants at the time of enrollment. However, seven subjects indicated past treatment with selective serotonin reuptake inhibitors (SSRIs). Additionally, subjects who reported a psychological disorder other than PTSD as the primary cause for distress or those who identified an index trauma that was not combat-related were excluded from the study. Exclusion criteria for the current study were meant to limit possible confounding variables that may influence emotional processing and or CV health, but to not exclude some of the more common comorbidities associated with PTSD (e.g., depression, other anxiety disorders).

**Table 1:** Descriptive statistics.

	**Mean**	***SD***	**Median**	**MAD**
Age	32.4	7.2	31.0	5.9
Education	15.4	1.9	16.0	2.2
Smoking status	8.3% (*N* = 2)	–	–	–
Body mass index (BMI)	28.9	4.6	28.5	4.5
Basal systolic blood pressure	123.4	9.8	123.5	10.3
Basal diastolic blood pressure	78.9	10.5	79.5	6.7
Basal heart rate	65.3	10.2	66.5	9.6
Physical activity level (METs)	8,198	9,694.7	3,240	4,269.9
Life events checklist	6.5	2.5	7.0	3.0
Combat and post battle experiences	15.8	6.3	17.0	8.2
Total duration of combat exposure (months)	18.3	13.6	12.5	8.1
Depression severity	9.3	7.6	8.5	6.7
Current PTSD severity	35.4	14.4	34.0	20.0
Lifetime PTSD severity	57.5	32.6	61.5	35.6
FMD (% dilation)	4.8	2.4	4.7	2.5
Total GM volume (mm^3^)	716,161.5	48,182.7	725,738.7	30,110.8
Left posterior insula volume (mm^3^)	1,275.2	213.2	1,270.5	188.3
Right posterior insula volume (mm^3^)	1,397.3	208.6	1,380.0	134.2
Left rostral ACC volume (mm^3^)	5,063.9	525.2	4,943.5	518.2
Right rostral ACC volume (mm^3^)	5,573.1	772.9	5,498.0	753.2

*SD, standard deviation; MAD, median absolute deviation; METs, metabolic equivalent; PTSD, posttraumatic stress disorder; FMD, flow mediated dilation; GM, Gray Matter; ACC, Anterior Cingulate Cortex*.

### Procedures

This study was approved by the University of Kansas Medical Center and the University of Missouri-Kansas City Institutional Review Boards. All subjects provided written informed consent prior to completion of the study protocol, in accordance with the Declaration of Helsinki. Participation involved a clinical, CV and neuroimaging assessment (described below). The clinical and neuroimaging (MRI) assessments occurred within approximately 2 weeks of each other, while the CV assessment occurred within 6 months of MRI acquisition.

#### Clinical assessment

The Mini-International Neuropsychiatric Inventory for DSM-IV (Sheehan et al., 1998) was used to assess Axis I disorders. PTSD symptom severity and diagnosis were assessed using the Clinician Administered PTSD Scale (CAPS)-IV (Blake et al., 1995). Given the variable time between CAPS and CV assessment, we used lifetime PTSD severity as the primary variable of interest. Thirteen veterans met diagnostic criteria for lifetime PTSD (when symptoms were most distressing or impairing). In contrast, only four met for current PTSD diagnosis (within the last 30 days of assessment). The Beck Depression Inventory-II (Beck et al., 1996) was used to assess depressive symptomology. To assess all traumatic experiences associated with combat, we computed a composite score of the Combat (e.g., encountering an explosive device) and Post Battle Experiences (e.g., handling human remains) subscales from the Deployment Risk and Resiliency Inventory (King et al., 2006).

#### Neuroanatomical assessment

Scanning was conducted on a Siemens 3.0 Tesla Skyra MRI scanner. To minimize susceptibility artifact in ventromedial prefrontal regions and to standardize head positioning, subjects were positioned so that the angle of the AC-PC plane was between 17° and 22° in scanner coordinate space. A T1-weighted magnetization prepared rapid gradient echo (MPRAGE) anatomical scan was acquired using a 3D MPRAGE sequence (*TR*/*TE* = 2,300/2 ms, flip angle = 8°, FOV = 256 mm, matrix = 256 × 256, 1 mm^3^ voxels) and used for volumetric analyses.

**Figure 1:**
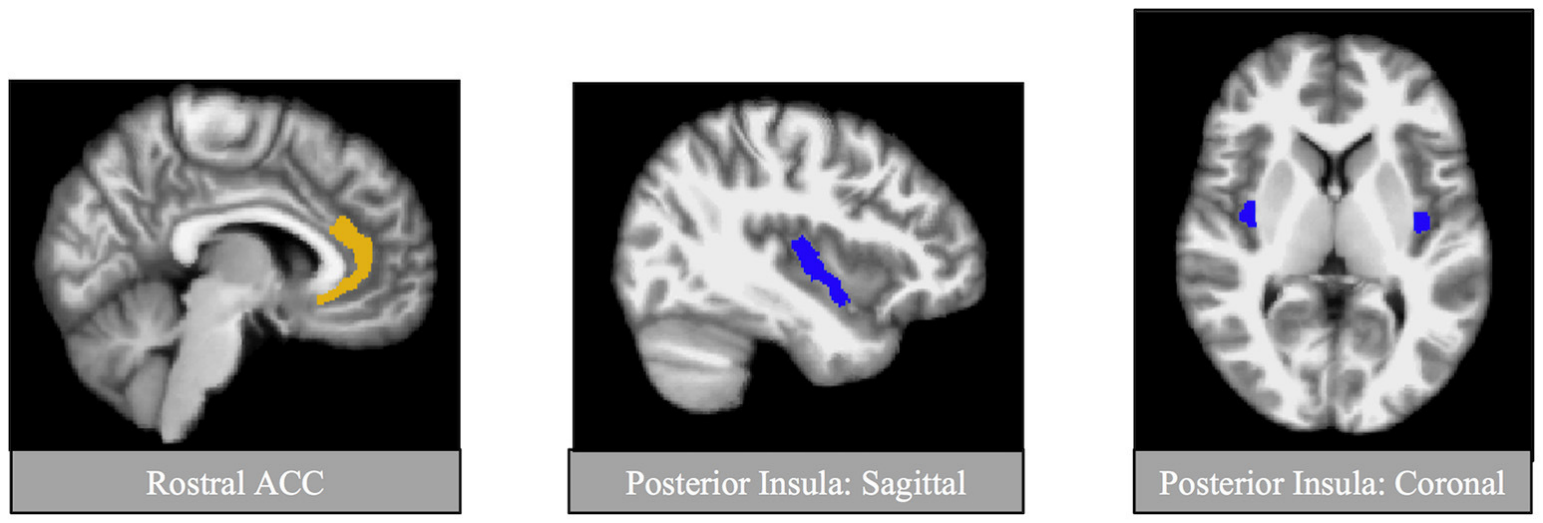
Regions of interest for primary analyses. Regions of interests were identified using automatic segmentation (Fischl et al., 2002) through FreeSurfer image analysis suite (available at: http://surfer.nmr.mgh.harvard.edu/). Shown here are the primary regions of interest, the posterior insula and the rostral anterior cingulate cortex (ACC).

All images were preprocessed using FreeSurfer version 5.1.0 image analysis suite (http://surfer.nmr.mgh.harvard.edu/). Quality assurance (QA) and manual editing were conducted by a trained rater (ANC). Briefly, QA included assessment of volumetric outliers (none present in the current sample) and visual inspections of registration of the image to the Talairach atlas template, skull stripping, and white matter and pial surfaces. If necessary, manual edits were made to correct FreeSurfer's performance. All scans passed QA and were therefore included in the analyses. Bilateral and whole brain volumetric estimates were extracted from individual anatomical images. The primary regions of interest (ROIs) were the rostral ACC and posterior insula (Figure 1). GM volume for each region was compute relative to whole brain GM volume. Additionally, we conducted exploratory analyses to explore GM volume within the anterior insula, hippocampus, and amygdala given that these regions are also implicated in both PTSD and CV regulation (Gilbertson et al., 2002; Sanders et al., 2003; Wignall et al., 2004; Karl et al., 2006; Paulus and Stein, 2006; Shin et al., 2006; Gianaros et al., 2008; Garfinkel and Liberzon, 2009; Woon et al., 2010; Li et al., 2014). ROI's were identified based on FreeSurfer's anatomical labeling (Destrieux et al., 2010). The anterior part of the cingulate gyrus and sulcus (Index 6), and the long insular gyrus and central sulcus of the insula (Index 17) were used to estimate regional GM volume of the rostral ACC and posterior insula, respectively. ROI's are depicted in Figure 1.

#### Cardiovascular assessment

Subjects completed the Global Physical Activity Questionnaire (GPAQ; Armstrong and Bull, 2006) to assess physical activity. The GPAQ yields a total score of metabolic equivalents (METs) in which higher scores indicate higher levels of activity. Scores > 600 METs indicate subjects meeting World Health Organization's recommendations on physical activity for health. Height and weight were obtained to calculate body mass index (BMI). Basal blood pressure and heart rate were also obtained in the right arm following a 5-min resting period. FMD methodology was based on previously published procedures (Billinger et al., 2012, 2017; Kluding et al., 2015; Clausen et al., 2016). We used an 8.0 Mhz linear array probe attached to a high-resolution ultrasound machine (Acuson, Sequoia 512, Siemens Medical Solutions USA, Inc., Mountain View, CA). For this procedure, a blood pressure cuff was placed immediately distal from the olecranon process (elbow). Subjects were asked to lie in the supine position for 20 min. At 19 min, a baseline blood pressure was obtained. At 20 min, baseline brachial artery images were obtained and recorded for a 60-s period to establish a baseline diameter, pre-occlusion. The blood pressure cuff was inflated to 200 mmHg for 5 min. A stereotactic clamp was used to stabilize the ultrasound transducer and hold it in place during the procedure. Images were recorded 20 s prior to cuff deflation for a total of 3-min. This procedure was conducted in a temperature and humidity controlled room. Scans were performed between 7 and 9 a.m. after an overnight fast to minimize the effects of diet and medication. FMD images were analyzed using specialized software (Brachial Analyzer, Medical Imaging Applications, Coralville, Iowa).

### Statistical analyses

Robust, non-parametric analyses were employed (using R statistical package; cran.r-project.org) to limit the impact of non-normally distributed data and potential outliers, and to accommodate the small sample size (Wilcox, 2012). Age and education were explored as covariates. Relationships of rostral ACC and posterior insula volume with (1) combat experiences, and (2) FMD were tested using Theil-sen regression analyses, which computes median of all possible slopes between the predictor and outcome (Wilcox, 2012). Results were corrected for multiple comparisons using a Holm-Bonferroni adjustment, resulting in the corrected critical *p* < 0.025 and 0.05 for each hypothesis (two regressions per hypothesis). While our primary hypotheses focus on combat experiences due to results from previous research (Clausen et al., 2016), we also explore relationships with PTSD symptoms and GM volume using Theil-sen regressions. Analyses examining intersecting relationships of combat experiences (independent variable) with FMD (outcome variable), and GM volume (mediator) of ROIs identified in regression analyses were tested using Sobel mediation (Sobel, 1982) with bootstrapping (with replacement; *N* = 2,000). While primary analyses focused on the average of bilateral GM volumes, parallel *post-hoc* analyses were conducted to determine potential influence of laterality for ROI's significantly related to combat and or FMD.

We also explored relationships parallel to those described above for secondary ROIs including the anterior insula, amygdala, and hippocampus. Given that the research related to the secondary ROIs is less consistent, we did not identify an *a priori* hypothesis. For these exploratory analyses, results were considered significant at an alpha-level of 0.05.

## Results

Descriptive statistics are presented in Table 1. Education and age were not related to posterior insula and rostral ACC volume or FMD (all *p*'s > 0.10) and, thus, were not included as covariates in primary analyses. Additionally, conventional risk factors associated with cardiovascular disease including systolic and diastolic blood pressure and heart rate were within normal limits (Whelton et al., 2017) and did not relate to regional GM volume or FMD (all *p*'s > 0.10). While regional GM volume was corrected for total (cortical and subcortical regions) GM volume (regional GM volume/total GM volume), total GM volume did not relate to PTSD symptoms or FMD and was therefore not included as a covariate in statistical analyses.

**Figure 2:**
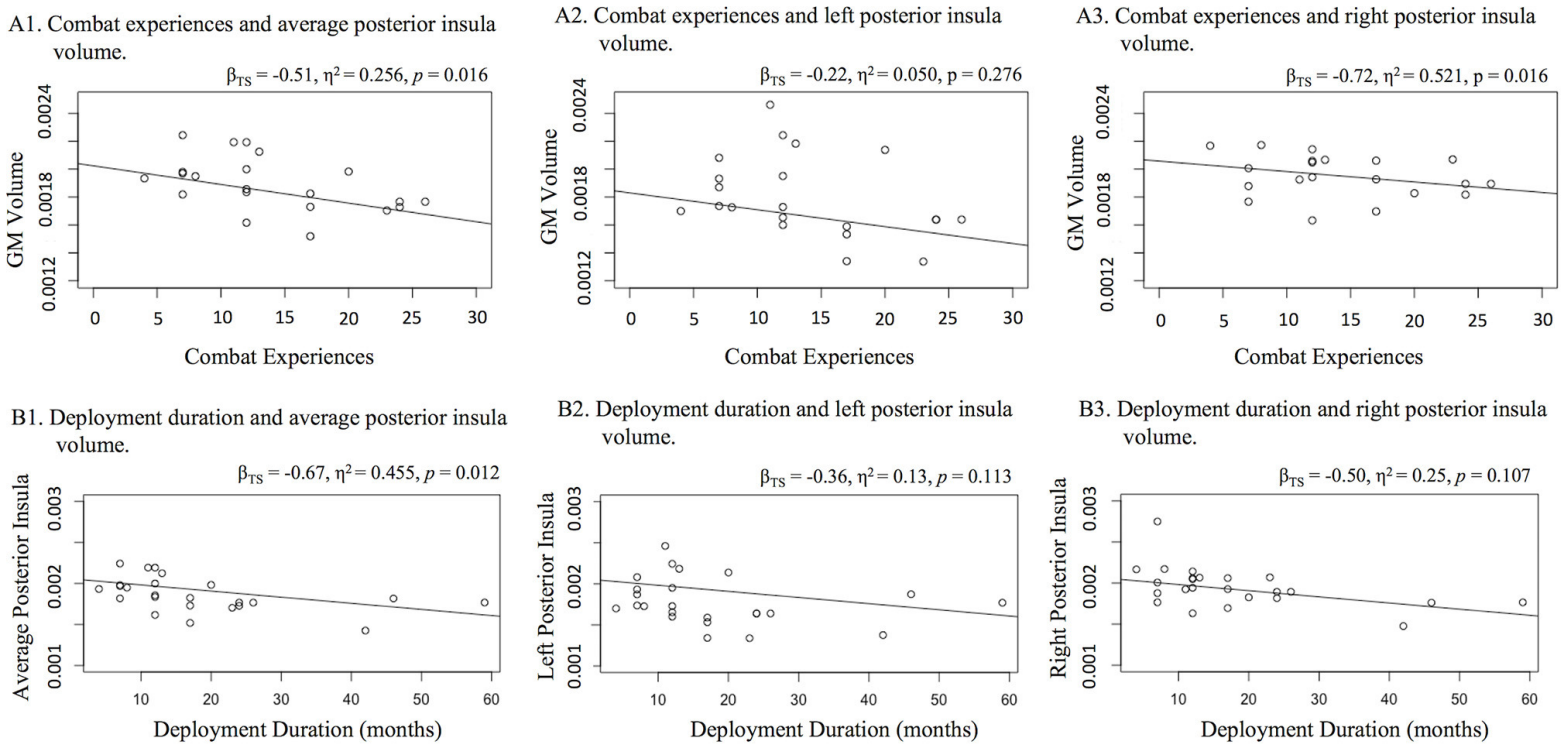
Relationships with posterior insula volume **(A)** combat and **(B)** deployment duration. Figure 1 displays three scatter plots of relationships with **(A)** combat experiences and **(B)** deployment duration with average bilateral, left, and right posterior insula gray matter volume. Increased number of combat experiences and increased deployment duration related to lower average posterior insula volume. Bilateral investigation suggests that these relationships may have been more robust within the right hemisphere.

A significant and large effect of combat experiences was found on posterior insula volume (β_TS_ = −0.51, η^2^ = 0.256, *p* = 0.016; Figure 2). This finding was most robust for the right posterior insula (β_TS_ = −0.72, η^2^ = 0.521, *p* = 0.016), while this relationship was not significant for the left posterior insula (β_TS_ = −0.22, η^2^ = 0.050, *p* = 0.276).

To specify the impact of combat exposure, we examined whether the number of deployments and deployment duration were associated with posterior insula volume. A Theil-sen regression was used to assess number of deployments and posterior insula volume. A Welch's one-way analysis of variance (trimmed means of 0.2) was used to explore potential differences in posterior insula volume between veterans with multiple vs. single deployments. Veterans endorsing multiple (vs. single) deployments exhibited significantly smaller posterior insula volume [*W*_(1, 10.39)_ = 12.38, *p* = 0.005]. Also, increased deployment duration was related to smaller average posterior insula volume (β_TS_ = 0.67, η^2^ = 0.455, *p* = 0.012; Figure 2). Increased PTSD severity was related to smaller posterior insula volume (β_TS_ = −0.49, η^2^ = 0.240, *p* = 0.026) with a moderate to large effect, but this did not survive a Holm-Bonferroni adjustment (*p* < 0.025). PTSD symptom severity was significantly related to lower volume in the right posterior insula (β_TS_ = −0.59, η^2^ = 0.351, *p* = 0.047), yielding a large effect. However, PTSD symptom severity was not related to lower volume within the left posterior insula (β_TS_ = −0.30, η^2^ = 0.090, *p* = 0.223).

Combat experiences (β_TS_ = 0.06, η^2^ = 0.004, *p* = 0.862), the number of deployments [*W*_(1, 10.03)_ = 0.16, *p* = 0.70], deployment duration (β_TS_ < 0.001, η^2^ < 0.001, *p* > 0.99) and PTSD severity (β_TS_ = −0.11, η^2^ = 0.013, *p* = 0.69) were not relate to rostral ACC volume. In addition, GM volume of secondary ROIs (anterior insula, amygdala, and hippocampus) were not related to combat experiences, number of deployments, deployment duration nor PTSD severity in the present sample (*p* > 0.10).

Combat experiences were marginally related to FMD (β_TS_ = −0.30, η^2^ = 0.089, *p* = 0.091), but the relationship did not meet statistical significance. FMD was also not related to rostral ACC (β_TS_ = 0.07, η^2^ = 0.006, *p* = 0.749) or posterior insula (β_TS_ = 0.14, η^2^ = 0.020, *p* = 0.572) volumes. As neither combat experiences nor FMD related to rostral ACC volume, mediation analyses focused on the posterior insula. However, present results do not support an indirect effect of posterior insula volume on the relationship between combat experiences and FMD (β = −0.060, 95% *CI* = −0.2269, 0.1703).

## Discussion

Trauma exposure (e.g., combat), as well as a specific diagnosis of PTSD, increase the risk for developing CV disease (Elder et al., 1997; Wentworth et al., 2013) and CV-related death (Boscarino, 2008). Brain regions including the ACC and the insular cortex have been implicated in both PTSD and CV regulation (Oppenheimer et al., 1992; Kasai et al., 2008; Herringa et al., 2012), suggesting that these regions may contribute to the increased prevalence of CV disease in combat veteran populations. The present study explored the intersecting relationships of combat experiences, CV health, and rostral ACC and posterior insula volume, in a sample of relatively young and physically healthy veterans. Results provide initial evidence that posterior insula volume relates to level of combat experiences, as well as the number and duration of deployments. Additionally, while not statistically significant, we found a moderate effect between increased combat experiences and poorer FMD response. However, results do not provide support for our hypothesis that posterior insular volume would mediate the relationship between combat experiences and CV health.

### Combat experiences, PTSD symptomology, and GM volume

Previous neuroimaging studies in trauma-exposed populations have concentrated on the anterior insula, which is implicated in the integration of information about salience and emotion with information concerning internal bodily states (Paulus and Stein, 2006). This body of research suggests that individuals with PTSD exhibit smaller anterior insula volume compared to those without PTSD (Chen et al., 2006; Kasai et al., 2008; Herringa et al., 2012). However, the posterior insula, implicated in processing visceral information (Craig, 2002) and regulation of the autonomic nervous system (Oppenheimer et al., 1992), has received less attention. Given the autonomic dysregulation commonly associated with combat experiences and PTSD (Friedman and Schnurr, 1995), the posterior insula was a prime target for our study. Results provide initial evidence that greater levels of combat experiences and deployment duration relate to smaller posterior insula volume. Additionally, while the present results did not meet statistical significance, a large effect was also found for PTSD symptom severity in relation to smaller posterior insula volume. Moreover, compared to those with single deployment, veterans with two or more deployments exhibited significantly smaller posterior insula volume indicating that smaller volume might be exacerbated by exposure to multiple stressors. These relationships were more robust within the right hemisphere. While there is limited research related to posterior insula and combat experiences, brain stimulation research in individuals with epilepsy suggests that the right posterior insula is associated with tachycardia, and pressor (increased systolic blood pressure) responses; whereas the left posterior insula is associated with bradycardia and depressor (increased diastolic blood pressure) responses (Oppenheimer et al., 1992). Therefore, present results may suggest that combat experiences are linked more closely with pressor responses such as hyperarousal. However, future research probing both pressor and depressor systems will be beneficial for further clarify these relationships.

Prior research has consistently demonstrated a relationship between PTSD and GM volume within the rostral ACC, and to a lesser extent within the anterior insula, amygdala, and hippocampus (for review, see Stillman and Aupperle, 2015). However, in the present study combat experiences were not related to any of these regions. It is possible that these volumetric differences are most evident in populations with more chronic or more severe levels of PTSD than endorsed in the present sample. Furthermore, intervention research examining the potential effects of psychotropic medication on GM volume suggests that SSRIs may beneficially impact GM volume (Vermetten et al., 2003), particularly within the hippocampus. This may serve as a confounding variable in the present study as seven veterans had previously taken SSRIs.

### Cardiovascular health and GM volume

FMD was not associated with rostral ACC and posterior insula volume. There are several possible explanations for these null findings. First, as previously reported, veterans in the present sample exhibited poor FMD response (Clausen et al., 2016), with limited variability in FMD scores, which may have limited our ability to detect clinically significant relationships. Second, prior findings that poorer CV health is linked to reduced or smaller whole brain volume have been reported in older adults (Ward et al., 2005; Jefferson et al., 2007; Hillman et al., 2008; Ho et al., 2010; Muller et al., 2011; Bosi et al., 2012). However, this is the first study to examine the relationship between CV health and regional GM volume in a young adult sample. Thus, it is possible that the relationships with GM volume and chronic CV health problems may only be seen after a more prolonged period or after there has been chronic CV dysfunction to lead to more significant atrophy (Muller et al., 2011; Bosi et al., 2012). Alternatively, it is possible that CV disease may relate more directly to changes in white matter integrity (Bijanki et al., 2013), rather than changes in GM volume. Lastly, the relatively high level of physical activity endorsed in the present sample may be serving as a protective factor against GM volume reductions (Colcombe et al., 2006; Erickson et al., 2009). Longitudinal studies will play a vital role in clarifying the relationships of combat experiences and PTSD with GM volume and CV health. Future research is also needed to investigate other potential paths linking PTSD to CV dysregulation, including white matter integrity. Additionally, future research is warranted to explore multiple measures of CV health (e.g., markers of inflammation), in order to determine the unique and combined influence of each proposed pathway.

### Limitations

One limitation of the current study was a relatively small sample size (*N* = 24), as well as moderate levels of PTSD symptomology, which may lead to low statistical power, impacting our ability to detect significant relationships with small to moderate effects. The lack of a non-combat-exposed group is another limitation, such that it remains unclear whether there are differences in rostral ACC/posterior insular volume and FMD between individuals with and without combat exposure. Furthermore, intervention research examining the potential effects of psychotropic medication on GM volume suggests that selective serotonin reuptake inhibitors (SSRIs) may beneficially impact GM volume in PTSD patients (Vermetten et al., 2003), particularly within the hippocampus. The present study included seven veterans who had previously taken SSRIs, which might result in confounding effects. Additionally, we focused exclusively on FMD as an estimate of CV health, which represents only one aspect of CV disease. Lastly, a cross-sectional design limits the ability to delineate temporal relationships. Future research is also needed to (a) investigate other potential variables mediating or moderating trauma exposure and CV dysregulation (e.g., white matter integrity, brain activation) and (b) assess multiple measures of CV health (e.g., markers of inflammation) to determine the mechanisms underlying the effects of trauma on CV health.

## Conclusions

Despite the limitations, results from the present study provide an initial framework to examine intersecting relationships of combat experiences, CV health (as measured by FMD), and GM volume in a sample of relatively young, and physically healthy combat veterans. Increased PTSD symptom severity and increased combat experiences were related to smaller posterior insula volume, particularly within the right hemisphere. FMD was not related to regional GM volume within the rostral ACC or the posterior insula as hypothesized. Future longitudinal research is warranted to explore the relationships of PTSD with CV health and brain morphology in a larger, more diverse populations, and with other measures of CV health (e.g., inflammation markers, inter-arterial plethysmography) to more clearly understand these intersecting relationships.

## Author contributions

AC: Substantial contributions to the conception and design of the work; data acquisition, data analysis and interpretation of results. Contributions to drafting, and revising the work critically for important intellectual content. Provided final approval of the version to be published, and agreement to be accountable for all aspects of the work in ensuring that questions related to the accuracy or integrity of any part of the work are appropriately investigated and resolved. SB: Substantial contributions to the conception and design of the work. Revising the work critically for important intellectual content. Provided final approval of the version to be published, and agreement to be accountable for all aspects of the work in ensuring that questions related to the accuracy or integrity of any part of the work are appropriately investigated and resolved. J-FS Substantial contribution to data acquisition. Revising the work critically for important intellectual content. Provided final approval of the version to be published, and agreement to be accountable for all aspects of the work in ensuring that questions related to the accuracy or integrity of any part of the work are appropriately investigated and resolved. HS: Substantial contribution to data analysis and interpretation of results. Revising the work critically for important intellectual content. Provided final approval of the version to be published, and agreement to be accountable for all aspects of the work in ensuring that questions related to the accuracy or integrity of any part of the work are appropriately investigated and resolved. RA: Substantial contribution to the concept or design of the work, and interpretation of results. Revising the work critically for important intellectual content. Provided final approval of the version to be published, and agreement to be accountable for all aspects of the work in ensuring that questions related to the accuracy or integrity of any part of the work are appropriately investigated and resolved.

### Conflict of interest statement

The authors declare that the research was conducted in the absence of any commercial or financial relationships that could be construed as a potential conflict of interest.
